# Supplementary material for: The interactome of intact mitochondria by cross-linking mass spectrometry provides evidence for coexisting respiratory supercomplexes
Source: Mol Cell Proteomics. 2017 Dec 8;17(2):216–32. doi: 10.1074/mcp.RA117.000470 (PMC5795388; doi:10.1074/mcp.RA117.000470)
Supplement: Supplemental Data [file supp_RA117.000470_133922_0_supp_23978_fzffwf.zip › spectra_annotation/mito_spectra_annotation/123-1-9-1-72-1.pdf]

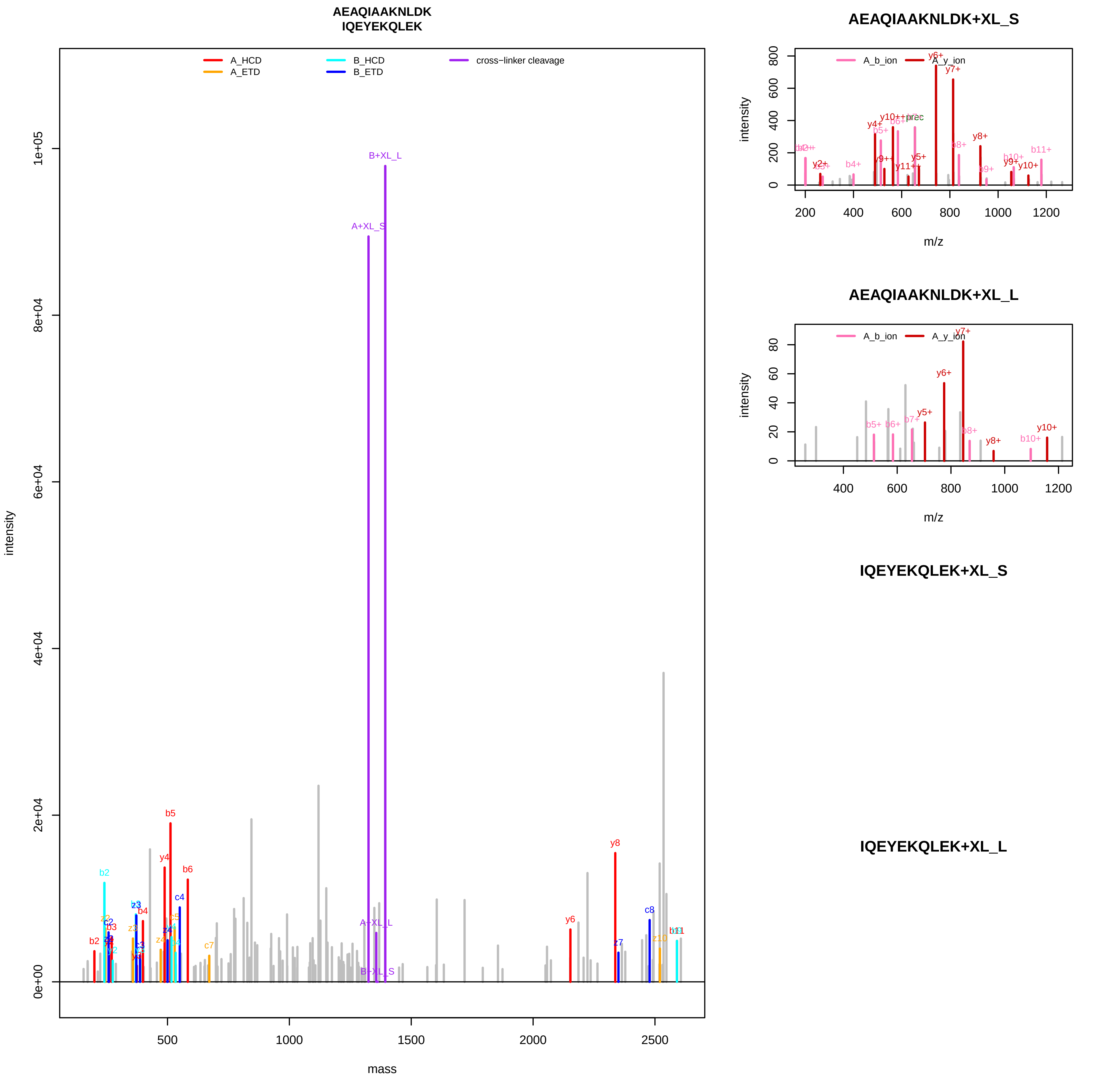
AEAQIAAKNLDK
IQEYEKQLEK
A_HCD
A_ETD
B_HCD
B_ETD
cross−linker cleavage
B+XL_L
A+XL_S
A+XL_L
B+XL_S
intensity
mass
0e+00
2e+04
4e+04
6e+04
8e+04
1e+05
500
1000
1500
2000
2500
b2
b3
b4
b5
b6
y4
y6
y8
c4
c7
c8
z7
b11
AEAQIAAKNLDK+XL_S
A_b_ion
A_y_ion
y6+
y7+
y8+
b8+
b11+
y10+
m/z
AEAQIAAKNLDK+XL_L
y7+
y6+
y5+
y10+
b10+
IQEYEKQLEK+XL_S
IQEYEKQLEK+XL_L
